# The Genetic Regulation of Aortic Valve Development and Calcific Disease

**DOI:** 10.3389/fcvm.2018.00162

**Published:** 2018-11-06

**Authors:** Vinal Menon, Joy Lincoln

**Affiliations:** ^1^Center for Cardiovascular Research, The Research Institute at Nationwide Children's Hospital, Columbus, OH, United States; ^2^The Heart Center, Nationwide Children's Hospital, Columbus, OH, United States; ^3^Department of Pediatrics, Ohio State University, Columbus, OH, United States

**Keywords:** aortic valve calcification, extracellular matrix, valve interstitial cell, valve endothelial cell, hemodynamics, epigenetics, signaling, development

## Abstract

Heart valves are dynamic, highly organized structures required for unidirectional blood flow through the heart. Over an average lifetime, the valve leaflets or cusps open and close over a billion times, however in over 5 million Americans, leaflet function fails due to biomechanical insufficiency in response to wear-and-tear or pathological stimulus. Calcific aortic valve disease (CAVD) is the most common valve pathology and leads to stiffening of the cusp and narrowing of the aortic orifice leading to stenosis and insufficiency. At the cellular level, CAVD is characterized by valve endothelial cell dysfunction and osteoblast-like differentiation of valve interstitial cells. These processes are associated with dysregulation of several molecular pathways important for valve development including Notch, Sox9, Tgfβ, Bmp, Wnt, as well as additional epigenetic regulators. In this review, we discuss the multifactorial mechanisms that contribute to CAVD pathogenesis and the potential of targeting these for the development of novel, alternative therapeutics beyond surgical intervention.

## Introduction

**Figure 1:**
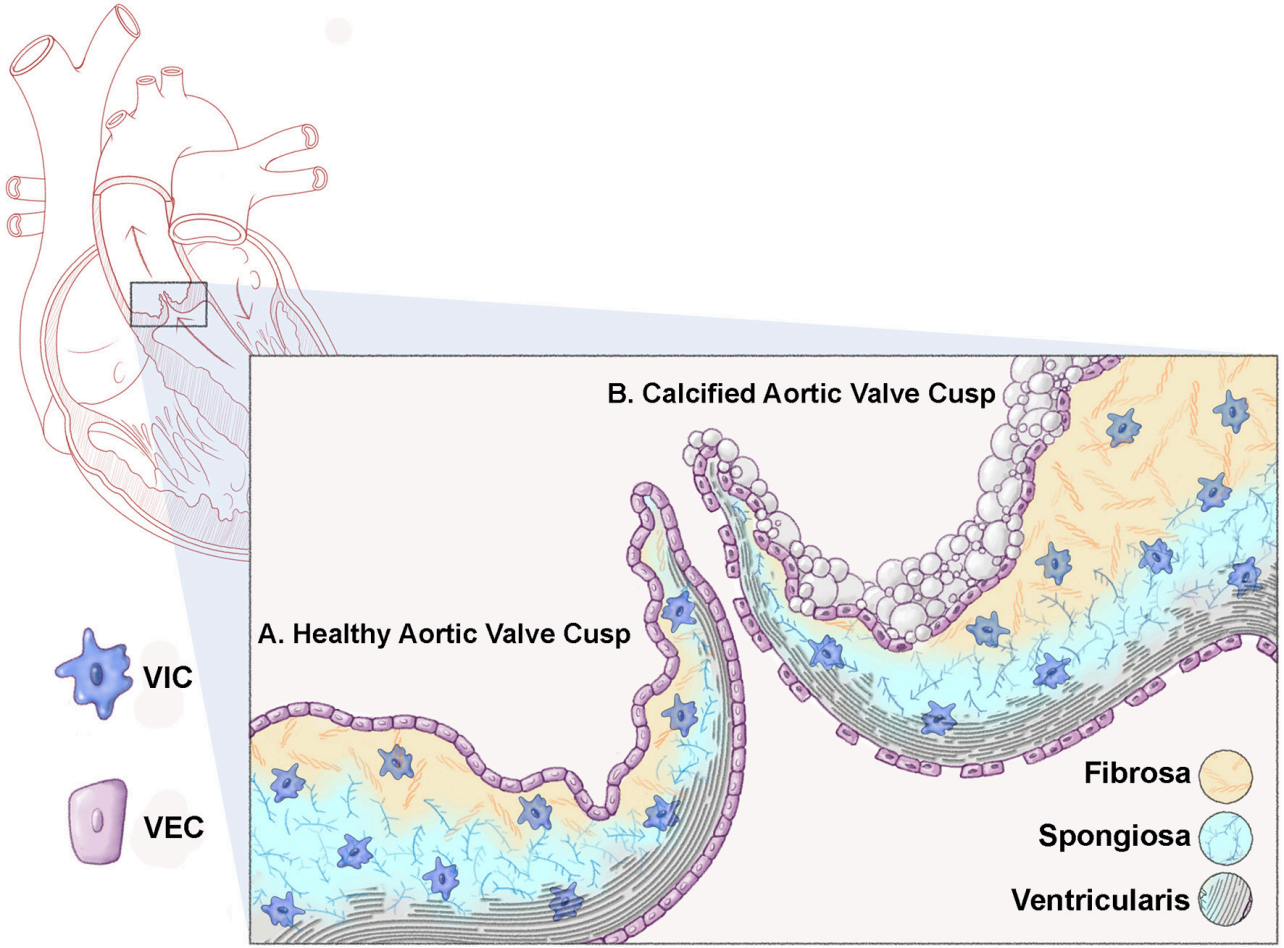
Schematic representation of a healthy and calcified aortic valve cusp. Cross sectional representation of a heart highlighting the aortic valve (box). **(A)** A healthy aortic valve cusp structure consists of three layers of extracellular matrix (ECM); the ventricularis (elastin fibers, black); the spongiosa (proteoglycans, blue); and the fibrosa (collagens, yellow). In addition to the matrix, the valve cusp is encapsulated by a single layer of valve endothelial cells (VECs), while valve interstitial cells (VICs) populate the core of the cusp. **(B)** A calcified valve cusp is thickened with abrogations in ECM organization including fragmented elastin fibers and increased collagen content. In addition, calcific nodules form on the fibrosa surface of the cusp (off-white).

Two sets of cardiac valves open and close over 100,000 times a day to maintain unidirectional blood flow through the heart. The atrioventricular (AV) valves (mitral, tricuspid) regulate flow from the atria into the ventricular chambers, while the semilunar valves (aortic, pulmonary) guide flow out of the ventricles into the pulmonary and systemic circulation. This necessitate function of the valves is largely facilitated by a highly organized connective tissue, composed of stratified layers of extracellular matrix (ECM), and valve interstitial and endothelial cell populations that molecularly communicate (Figure 1A). Establishing and maintaining heart valve connective tissue is essential for structure-function relationships, and aberrations in embryonic development or adult homeostasis underlie dysfunction and disease that affect more than 5 million Americans each year (1, 2).

In contrast to healthy valve structures, diseased valves are characterized by loss of ECM organization and injury to, or dysfunction of valve cell populations (1). Calcific aortic valve disease (CAVD) is the most common form of valvular pathology and the third most common cardiovascular disease following hypertension and ischemic heart disease (3–5). The disease is a significant economic and healthcare burden contributing to over 15,000 deaths in the US annually (6). The pathogenesis of affected aortic valves is characterized by ectopic development of calcium nodules on the aortic surface of the valve cusp, annulus, or both (Figure 1B). The resulting sclerotic aortic valve exhibits compromised function by virtue of limited movement which progresses to calcific aortic stenosis over time (7). Over 2.5 million individuals are affected by aortic stenosis which can lead to significantly impaired cardiac function and if left untreated, death. CAVD and aortic stenosis is most prevalent in the aging population but is also observed during earlier stages of life in individuals born with bicuspid aortic valve (BAV) disease.

The underlying etiology of CAVD is largely unknown. While BAV is proven hereditary, there is limited information regarding genetic contributions to CAVD [reviewed in (8)]. Risk factors are known and similar to most other cardiovascular diseases (hypertension, tobacco-use, hypercholesterolemia, etc.), however the mechanisms that underlie calcific nodule formation on the aortic cusp surface following exposure to risk factors are largely unknown. The hemodynamic environment of the aortic valve is known to play a role in valve pathogenesis, and studies using animal and *in vitro* models have identified aberrations in critical signaling pathways required for valve formation in CAVD [reviewed in (8)]. However, the field has yet to delineate cause and effect of these multifactorial contributors. The current limitations in understanding the etiology of CAVD has hindered the development of alternative therapeutics beyond surgery, to prevent or regress CAVD. Therefore, further basic science research is needed to decipher the cellular and molecular processes underlying the pathology of CAVD and translate these discoveries into mechanistic-based pharmacological therapies to reestablish valve structure-function relationships.

## Healthy heart valve structure-function relationships

The mature valve structures are composed of leaflets (AV) or cusps (semilunar) with supporting structures. In the AV position, the mitral valve consists of two leaflets, while the tricuspid possesses three, and both display external supporting chordae tendineae that attach the underside of the valve leaflet to the papillary muscles within the ventricle (9). The three cusps of the semilunar valves (aortic, pulmonic) lack external support, but a unique supporting structure within the aortic roots in the form of a fibrous annulus has been described (9). The “Lub-Dub” noise of the heart beat is attributed to sequential closing of the AV and semilunar valve leaflets/cusps, respectively, during the cardiac cycle and this is driven by the valve hemodynamics. In systole, the aortic valve cusps open and experience oscillatory flow patterns on the aortic surface and laminar shear on the ventricular side with overall low stress, while the mitral valve leaflets are closed to prevent back flow into the left atrium and therefore pressure is high on the ventricular side. In contrast during diastole, the closed aortic cusps create high pressure and tensile stretch on the aortic and ventricular surfaces, respectively, while open mitral leaflets experience laminar shear flow and reduced pressure (10). This coordinated movement of the valve leaflets/cusps and their supporting structures in response to the hemodynamic environment is attributed to a highly specialized connective tissue that provides all the necessary biomechanical properties during diastole and systole. The extracellular component of the valve connective tissue is largely composed of three stratified layers of matrix arranged according to blood flow (see Figure 1A) (1, 11, 12). The cross-sectional structure of healthy valve leaflets contains the fibrosa layer located on the ventricular side of the AV valve leaflets and atrial side of the semilunar valves, away from blood flow. This layer is predominantly composed of bundles of collagen fibers aligned along the circumferential direction of the free edge of the leaflets (13–16). This arrangement provides tensile strength and flexibility to the valve leaflet/cusp during opening, while transmitting forces to promote coaptation of the leaflets in the closed position (17–19). Adjacent to the fibrosa is the spongiosa layer, with a lower abundance of collagens, high prevalence of proteoglycans, and water retention. This composition provides a more compressible matrix, allowing the valve to geometrically “flex” and absorb high force (16, 20). Finally, the layer adjacent to blood flow is termed the atrialis (AV) or ventricularis (semilunar) and largely consists of radially orientated elastin fibers that allow for high deformations to facilitate tissue movement as the valve leaflet opens and recoils during closure (21–23). In the mitral position, histological studies of human tissue report an additional fourth layer of elastin on the opposing side to the atrialis, which presumably allows for further flexibility (11). The AV chordae tendinae are composed of a cylindrical collagen core within an elastin sheath and exhibit high viscoelastic properties, while the “built-in” supporting structures of the semilunar valves contain similar extracellular matrix (ECM) components only arranged within the underside of the cusp structure (1, 24, 25). The overall protein contents of the valve matrix is adaptive and has been shown to remodel in response to normal wear-and-tear and aging and this is thought be beneficial in maintaining structure-function relationships throughout life (26).

In addition to the extracellular component of the valve, the mature leaflet/cusp contains several differential cell populations. The valve interstitial cells (VICs) are the most abundant cell type and have been described as heterogeneous and fibroblast-like in nature (27). In the healthy adult, VICs express markers such as Vimentin (28) and are considered quiescent as proliferation rates are comparatively low (~1%), functioning to mediate turnover of the valve ECM in response to general wear-and-tear (27, 29, 30). Previous studies have identified sub-populations of VICs based on molecular profiles (28), and this may be attributed to differential embryonic origins of this cell population (described below), or potential mechanical influences from the ECM or hemodynamic environment as described (31). Although VICs from the four heart valves all exhibit a fibroblast-like phenotype (32), those isolated from left-side valves (aortic and mitral) exhibit a greater stiffness compared to those isolated from right-side valves (33, 34). Furthermore, it was shown that aortic VICs exhibit greater capability of contracting the ECM relative to those isolated from pulmonary valves (34), highlighting the influence of the hemodynamic environment on VIC behavior.

In addition to VICs, the valve leaflet/cusp is encapsulated by a single layer of valve endothelial cells (VECs) that form a tight barrier between the circulating blood, and the underlying cellular and extracellular contents of the valve. VECs are dynamic, and molecular and phenotypic profiles are side-, and age-dependent (30, 35–38). Furthermore, there is increasing evidence to suggest that VEC function has important implications in regulating VIC behavior to maintain valve homeostasis and prevent disease (39–41). A relatively minor contributor to the valve cell population includes extra-cardiac hematopoietic or bone marrow-derived cells (42, 43). Under homeostatic conditions, these cells make up ~1–18% of the total valve cell population, depending on age and valve position, and maintain CD45 expression with co-expression of VIC markers including Vimentin (44). The function of these extra-cardiac cells in healthy valves remains elusive but numbers almost double in mouse models of valve disease (45, 46). Together, it's the integrated network of ECM components and valve cell populations that provide the necessary architecture for efficient valve function throughout life.

## Heart valve development

Formation of highly organized mature heart valves begins during embryogenesis when the primitive heart tube undergoes rightward looping and the cardiac jelly expands at the outflow tract (OFT) and the AV canal forming local swellings of endocardial cushions. At this time, a subset of endocardial cells overlying the cushions undergo endothelial-to-mesenchymal transformation (EMT) giving rise to a population of highly proliferative and migratory, valve precursor cells [reviewed in (47)]. EMT is a tightly regulated process important not only for valvulogensis, but embryonic development and differentiation in general. Early experiments employing the chick embryo as a model system demonstrated that explants of AV cushion consisting of endocardium and myocardium exhibited EMT when grown on collagen gels (48). Furthermore, there seems to be a need for specialized spatial signaling to induce EMT, as it was shown that only the endocardium of AV and OFT cushions, but not ventricular endocardium, undergoes EMT (49, 50). While the mechanisms of cushion development are conserved between AV and OFT valves, EMT in OFT cushions lags behind that occurring in the AV cushions. While endothelial-derived cells contribute the majority of mesenchyme cells within the cushions (51, 52), there is additional contribution from the cardiac neural crest and secondary heart field cells in the OFT position (53–55), and epicardial-derived cells are present in the parietal leaflets of the AV valves (56).

Many signaling pathways have been implicated in EMT and these cross-talk with each other to form complex molecular networks between differential cell types. The process is largely initiated by Transforming Growth Factor-β (Tgfβ) (47, 57–59), and Bone Morphogenetic Protein (BMP) (57, 60–68) signaling pathways emanating from the adjacent myocardium, as well as Notch predominant in valve endothelial cells (69–77). Wnt signaling in the endothelial-lineage is also critical for early stages of EMT (58, 78). Similar pathways (Tgfβ, Bmp, Wnt) also play a role in remodeling and sculpting the endocardial cushions as they morph into valve primordia during later stages of development (79–83). Aside from growth factors, the transcription factor Sox9 is required for proliferation of newly transformed mesenchyme cells and remodeling of the matrix, but not initiation of EMT (84, 85). In addition to molecular signaling, the hemodynamic environment is important for endocardial cushion formation and it is well-established that the endothelium, via mechanotransduction, senses and responds to hemodynamics (86) via the rearrangement of the cytoskeleton and alignment of these cells in a direction parallel to flow (87). *In vitro* studies employing a 3D tubular culture have identified the role of shear stress in the expression and deposition of fibrous ECM proteins in both AV and OFT cushions (88, 89). Furthermore, several *in vivo* studies reinforce the importance of maintaining normal hemodynamic stimuli during valve development, as demonstrated by the molecular/cellular responses that go awry when intracardiac hemodynamics are perturbed leading to a myriad of congenital heart defects (90–106). Defects in EMT or failure to establish the valve precursor cell pool during embryogenesis as a result of molecular abrogations or hemodynamic disturbances is lethal in mice and likely detrimental to human development. However, those affected by more subtle defects in post-EMT growth and maturation survive, but such disturbances could underlie valve malformations present at birth or acquired disease manifested later in life.

## Calcific aortic valve disease

Calcific aortic valve disease (CAVD) is the most predominant form of valve pathology affecting more than 5.2 million people in the US, particularly those over the age of 65 (107). In 2013, 50,222 deaths occurred due to valvular heart diseases in the USA, out of which 67.5% were due to aortic valve disorders (6). CAVD is an active cellular-driven pathological process beginning with alterations in the aortic valve cusps (sclerosis) and culminating in stenosis (108, 109). Risk factors for CAVD have been well-established and are shared with many other cardiovascular diseases (110). Histologically, sclerotic calcified valves are thickened with alterations in the composition and distribution of ECM components including fragmented elastin fibers and increased collagen fiber content (111). In addition, deposits of calcium and hydroxyapatite develop on the aortic surface of the cusp leading to bone-like, rigid nodules that limit cusp movement (Figure 1B) (112). The cellular mechanisms underlying the formation of calcific nodules within the valve tissue are not clear and likely diverse. Based on expression studies, it is considered that residing VICs are activated similar to myofibroblasts, and undergo transdifferentiation toward an osteoblast-like cell via a process similar to endochondral ossification. This is concluded from studies in calcified valves excised from humans that show ectopic expression of osteogenic transcription factors including Runx2, as well as mineralized matrix proteins (Matrix Gla Protein, Osteopontin, Bone Sialoprotein) commonly observed in bone (113–115). Histological analysis indicates that in CAVD, osteogenic-like changes (or increased Runx2) occurs in subset of VICs and not the entire population. Some attribute the pathological cell specificity to embryonic origin (116), others suggest that exogenous extra-cardiac cells play a role (117, 118), and there is no doubt that the hemodynamic environment influences formation of calcific nodules (119–121). While the field has been given a taster to these ideas, more in-depth studies are needed. In parallel with VIC pro-osteogenic fate changes in CAVD, studies have shown a significant contribution of extracellular vesicles (EVs) to the formation of calcific nodules at localized sites within the valve structure, similar to that observed in medial arterial calcifications and atherosclerotic intimal plaques (122), which suggest that the process of VIC differentiation and calcific nodule formation is multi-faceted.

CAVD is largely considered an acquired disease that develops later in life, however studies in mice might suggest that pathogenesis might stem from perturbations in embryonic development. Homozygous loss of *Notch1*, associated ligands or signaling mediators during gastrulation leads to embryonic lethality due to endocardial cushion defects as a result of defective EMT (69–77). Heterozygous mice are viable but when fed Western diet, develop CAVD (123), and cross breeding with the *Nos3*^−/−^ line increases this incidence with the additional development of BAV (40). In addition, *Notch1* is the most predominant disease-causing mutation in humans affected by calcification of the aortic valves (124). Similarly, loss of *Sox9* in endothelially-derived cells of the embryo causes early lethality by embryonic day (E)12.5 associated with hypoplasia of the endocardial cushions due to defects in proliferation of newly transformed mesenchyme cells following otherwise normal EMT (85). Despite this, heterozygotes have a normal lifespan, but develop CAVD phenotypes by 3 months of age, even without the addition of Western diet (85, 125). *In vivo* mutations, or targeted loss of function *in vitro* are sufficient to cause EMT defects [reviewed in (8)], and endothelial-specific deletion of *Tgf*β*1* promotes osteogenic differentiation of adult VICs and calcification (41). These studies suggest that *Notch1, Sox9*, and *Tgf*β*1* are required for valvulogenesis and viable mice with targeted reduced function develop valve disease later in life, potentially due to subtle embryonic defects that are manifested over time. BMP and Wnt are two signaling pathways previously shown to be activated in calcified valves from human patients, mouse models and cultured VICs (113, 126–137). However, mutations in Wnt or BMP signaling mediators have not been shown to be causative of CAVD, although tissue-specific deletion of the BMP receptor Alk2 underlies BAV in mice (138). The question beckons if “re-activation” of these developmental programs play a role in CAVD pathogenesis or if these are a read out of the pathological state.

**Figure 2:**
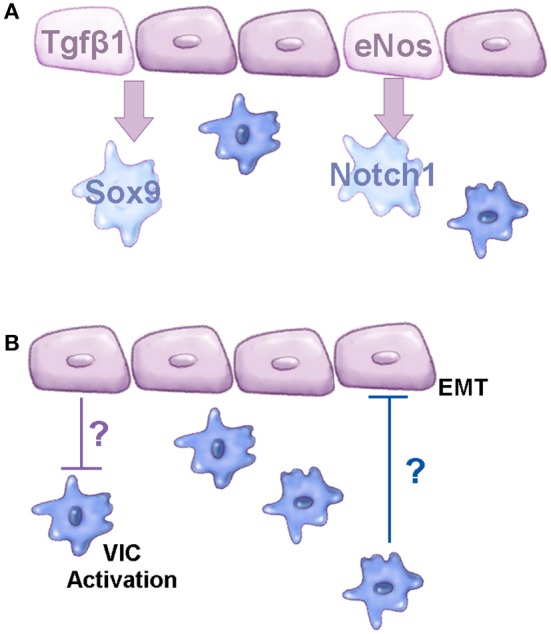
Molecular communications between VEC and VIC populations. **(A)** Schematic to show known signaling pathways active between VEC and VIC populations that prevent CAVD. **(B)** Diagram to demonstrate known cellular functions regulated by VEC-VIC communications; the mechanisms underlying these functions are currently unknown, based on studies by Hjortnaes et al. (140). Purple cells represent valve endothelial cells, blue cells are valve interstitial cells.

While several molecular pathways have been implicated in CAVD, the cellular mechanisms that underlie osteoblast-like changes in resident VICs have not been extensively explored. During early stages of CAVD, endothelial dysfunction has been reported (30, 139). In the vasculature, this pathological process has been well characterized, largely in the setting of atherosclerosis and plaque formation, however, in the valves, endothelial dysfunction is less well described. In a recent study, it was demonstrated that aging, a known risk factor of CAVD, is associated with reduced nitric oxide bioavailability (or increased reactive oxygen species) and decreased proliferation of VECs, in addition to enhanced permeability of the endothelial cell lining. Morphologically, VECs change in size and shape, and express distinct molecular profiles with aging (30). In the last 5 years, there have been an increasing number of studies to show that VECs are protective against calcific nodule formation by porcine aortic valve interstitial cells (pAVICs) *in vitro* and perturbations in VEC-VIC communication promote the onset of calcification (40, 41, 140). Protective paracrine factors emanating from VECs include nitric oxide (40) and Tgfβ1 (41) and their targets in VICs (and potentially VECs) have been identified as, but are not limited to *Notch1* (40) and *Sox9* (41), respectively (Figure 2A). There are likely many other pathways that exist between these two cell types that regulate cellular function. A previous study by Hjortnaes et al. demonstrated *in vitro* that VECs prevent activation of VICs through currently unknown mechanisms, while VICs prevent EMT of VECs suggesting two-way communications (Figure 2B) (140).

In addition to the molecular role of VECs communicating with underlying VICs, the endothelium is in direct contact with the circulation and it is known that VECs lining the ventricular and aortic cusp surface sense the complex, fluidic environment (35, 36, 39, 141, 142). There is also strong data to suggest that altered spatio-temporal flow patterns correlate with aortic valve disease. More specifically, it has been suggested that the local hemodynamic environment dictates the localization of calcified nodules, being present exclusively on the aortic aspect (fibrosa) of the aortic valve cusp (35, 143, 144). Furthermore, patients with bicuspid aortic valve (BAV) exhibit different hemodynamic profiles relative to that seen with tricuspid aortic valve (145, 146) and have an increased propensity to develop CAVD as early as 20–30 years of age (2, 147, 148). In addition to alterations in the hemodynamics surrounding the adult valve, studies have also demonstrated that altered intracardiac hemodynamics during early avian development perturbs key molecular pathways involved in valve development and disease, including Tgfβ signaling (100, 101, 106). While correlations between the valve hemodynamics and disease have been made, little is known about how mechanical stress on the endothelium translates into differentiation of VICs toward an osteoblast-like lineage. Previous studies have shown that the complex fluid mechanics surrounding the aortic valve is initially sensed by VECs lining the ventricular and aortic cusp surface (35, 36, 39, 141, 142). Compared to static flow conditions, unidirectional shear stress (20 dynes/cm) (or increased flow) promotes expression of inflammatory response genes and pro-calcification signaling, both of which are known contributors of CAVD pathogenesis (36, 39, 142). Interestingly, these pathogenic responses are greater in cells isolated from the aortic surface of the valve cusp, the side most susceptible to calcification (35, 36, 39, 142). With this in mind, it is appreciated how “endothelial cell dysfunction” has detrimental effects on relaying molecular cues to VICs and promoting pathogenic changes.

## Epigenetic regulation of aortic stenosis and calcific aortic valve disease

Dysregulation of critical signaling pathways are detrimental to valve cell function and structure-function relationships and there is emerging work to show that the epigenetic landscape can further influence gene regulation via multiple mechanisms including DNA methylation and non-coding RNAs (149, 150). Micro RNAs (MiRs) and long non-coding RNAs (LncRNAs) lack protein-coding function. MiRs are 18–26 bases long and predominantly inhibit expression of target genes either by directly preventing protein translation through target 3′-UTR binding or inducing mRNA degradation (151). In contrast, LncRNAs are more than 200 nucleotides long and can localize to different sub-cellular depots (152). While there are limited studies on the epigenetic regulation of cardiogenesis and valvulogenesis (153–155), gene methylation status, and LncRNAs and MiRs profiles have shown to be altered in CAVD and aortic stenosis and these are summarized in Tables 1, 2, respectively (181–183).

**Table 1:** The Role of DNA methylation in CAVD/Aortic Stenosis.

**Observation in CAVD/AS**	**Effect**	**Species**	**References**
Increased levels of *DNMT3B* in human stenotic aortic valves	Increase in global DNA methylation. More than 6,000 differentially methylated sites were identified between normal and stenotic valves. Expression of the osteogenic marker Sp7 was increased four-fold in stenotic vs. normal tissue	Human	(156)
Genetic inactivation of *DNMT3B*	Protects against activation of osteogenic pathways and slows the progression of aortic stenosis	Mouse	(157)
Altered CpG methylation in newborns with congenital aortic stenosis	Differentially methylated CpG sites	Human	(158)
Altered methylation of CpG sites	Contribution to regulation of left ventricular hypertrophy due to aortic stenosis -induced pressure overload	Human	(159)
Hypomethylation of LncRNA H19	Promotes VIC osteogenic-like changes by *NOTCH1* silencing	Human	(160)
5-methylcytosine (5meC) in intron 1 in a mammalian interspersed repeat element (MIR) was increased by 2.2-fold in CAVD compared to control aortic valves	*Phospholipid phosphatase* (*PLPP3*) gene and enzymatic activity were downregulated in mineralized aortic valves	Human	(161)
Decreased promoter methylation of the gene encoding the proinflammatory enzyme *5-lipoxygenase* (*5-LO*)	Increased *5-LO* mRNA levels. (Aortic stenosis is associated with increased leukotriene production, in part, due to induction of *5-LO* in VICs)	Human	(162)

**Table 2:** Role of non-coding RNAs in CAVD/Aortic Stenosis.

**Non-coding RNA**	**Mechanism in CAVD/AS**	**Target**	**Species**	**References**
LncRNA MALAT1	Upregulated. Positive regulator of osteodifferentiation by sponging miR-204	*miR-204*	Human	(163)
miR-214	Upregulation, increased fibrosa thickness and calcification were observed when porcine fibrosa was exposed to oscillatory shear.	*TGFβ1*	Porcine	(164)
LncRNA H19	Upregulated	*NOTCH1*	Human	(160)
miR-30c	Upregulated	*ITGB1, CXCL12, FLT1, CAMTA1, COL9A3*	Human	(165)
miR-486	Upregulated in TGFβ1 and BMP2-stimulated VICs and VICs from calcified aortic valves	*miR-204, Smurf2*	Human	(137)
miR-181b	Upregulated	*TIMP3, SIRT1, GATA6*	Human	(166)
miR-125b	Upregulated	*CCL4*	Human	(167)
miR-21-5p, miR-221-3p	Upregulated	*TGFβ, MAPK*, Wnt signaling pathways	Human	(168)
hsa-miR-193a-3p, hsa-miR-29b-1-5p, hsa-miR-505-5p, hsa-miR-194-5p, hsa-miR-99b-3p, and hsa-miR-200b-3p	Upregulated	Unknown	Human	(169)
LncRNA TUG1	Highly expressed. Sponges miR-204-5p	*miR-204-5p*	Human	(170)
miR-92a	Overexpressed in calcified bicuspid aortic valves	Unknown	Human	(171)
miR-204	Downregulated	*Smad4, Runx2*	Human	(137, 163, 172)
miR-141	Downregulated	*BMP2*	Porcine	(173)
miR-106a, miR-148a, miR-204, miR-211, miR-31 and miR-424	Downregulated	*Runx2, BMPR2, BMP2, BMP3, BMP8B, CBFB*	Human	(137, 163, 165)
miR-195	Downregulated	*Runx2, BMP2, Smad7, Smad1, Smad3, Smad5, Jag2*	Human	(174, 175)
miR-30b	Downregulated	*Smad1, Smad3, Runx2, caspase-3, Jag2, Smad7, Notch1*	Human	(174, 176)
miR-26a	Downregulated	*BMP2, ALP, Smad1, Jag2, Smad7, Runx2, Smad5*	Human	(174)
miR-122-5p	Downregulated	Lipid metabolism, *TGFβ*	Human	(168, 177)
miR-625-5p	Downregulated	Unknown	Human	(168)
miR-30e-5p	Downregulated	*PI3K-Akt*, MAPK signaling pathway	Human	(168)
hsa-miR-3663-3p, hsa-miR-513a-5p, hsa-miR-146b-5p, hsa-miR-1972, hsa-miR-718, hsa-miR-3138, hsa-miR-21-5p, hsa-miR-630, hsa-miR-575, hsa-miR-301a-3p, hsa-miR-636, hsa-miR-34a-3p, hsa-miR-21-3p, and hsa-miR-516a-5p	Downregulated	Unknown	Human	(169)
miR-10b	Downregulated	Inhibition of miR-10b in HL-1 cardiomyocytes caused over expression of *Apaf-1*	Human	(178)
miR-1, miR-133, miR-378	Downregulated	Unknown	Human	(179)
miR-616	SNP in *PON1* affects miRNA-mRNA interaction. Patients with the CT or TT genotype at loci rs3735590 were associated with a lower risk of CAVD than the patients harboring the CC genotype	*PON1*	Human	(180)

## Calcific aortic valve disease therapeutics; past, present, and future

Decalcification of aortic cusps with moderate aortic stenosis has been unsuccessful (184), and valve replacement surgery, either with mechanical or biological prostheses, is currently the treatment option of choice for CAVD patients. The ideal prosthetic valve would have hemodynamics similar to the native valve, grow and remodel with the individual, and not require additional therapy such as anticoagulation. However, to date we have yet to develop such an ideal prosthetic and there is a high rate of failure due to progressive deterioration including calcification and non-calcific damage that limits their effectiveness (185). This is likely related to the incompatibility of prosthetic valves with the native environment, leading to increased infiltration of inflammatory cells and disturbances in the hemodynamic environment (186, 187). Furthermore, the implanted valve will be similarly exposed to the underpinning risk factors of CAVD that were present before surgery. As a result, aortic stenosis patients are at risk of secondary left ventricular hypertrophy, and mortality rates of 2–3% have been reported (188–192). Transcatheter aortic valve replacement (TAVR) was pioneered in the early 2000s and is considered a less-invasive option for aortic stenosis patients (193, 194). Therefore, this procedure is common in patients deemed too “high risk” for conventional valve replacement surgery, which is advantageous for those suffering from senile-related CAVD. However, TAVR is also costly and not risk-free (195). In order to advance the field, tissue engineering approaches need to address the structural degeneration and thromboembolism risks of current prosthetics, and consider the need for growth and remodeling of implanted valves in the pediatric population. In parallel, there is a need to discover alternative therapeutics that go beyond surgery to halt or reverse the pathogenesis of CAVD.

Attempts have been made to pharmacologically treat CAVD. Statins, 3-hydroxy-3-methylglutaryl-coenzyme A reductase inhibitors, have been successfully used at reducing cardiovascular events (196, 197) by virtue of several pleiotropic beneficial effects including decreasing low-density lipoprotein (LDL) cholesterol, improving endothelial cell function, reducing inflammation, and decreasing thrombus formation (198). *In vitro* studies have shown pharmacological statins to be successful in curbing calcific nodule formation VICs (199), however, treatment with Simvastatin led to increased VIC osteoblast markers (200). The use of statins have been shown to successfully prevent CAVD in preclinical studies (201–204), however, these drugs do not seem to reduce CAVD or aortic stenosis in humans (205). Moreover, metabolic syndrome patients on statin treatment exhibited an increased rate of CAVD progression relative to placebo-treated control subjects (206). A major challenge in the use of statins for CAVD treatment is that the valve-specific molecular targets remain unclear, and hyperlipidemia/inflammation are not always causal of CAVD (207, 208). Furthermore, CAVD is often clinically detected as aortic stenosis, an advanced stage of the disease at which statins may not be useful in regressing or modulating pathology (208). Therefore, the utilization of statins in the treatment of CAVD remains unsubstantiated.

It has been shown that stenotic aortic valves have increased expression of the renin angiotensin (ag) system including ag-converting enzyme (ACE) and the type I ag II receptor (209). Animal studies have shown that ag-II receptor blocker (ARB) treatment inhibited sclerotic changes in the aortic valve, inhibited transdifferentiating of quiescent VICs to myofibroblasts and/or osteo-VICs while maintaining endothelial integrity (210). While ARBs are used to treat Marfan Syndrome (211), reports of therapeutic effects of ARBs for CAVD are mixed. It has been shown that ARBs could not prevent the progression of CAVD in elderly, high-risk hypertensive patients (212), however, other patients on ARBs had reduced aortic valve tissue remodeling (213) and, furthermore, treatment with ACE inhibitors and ARBs led to improved survival and a reduced risk of cardiovascular events in aortic stenosis patients (214), and might also abrogate CAVD pathogenesis in a hypertensive setting (215).

As discussed, the lncRNA and miRNA signatures are altered in the setting of CAVD, thus miRNA-based therapeutics maybe beneficial in restoring their expression and normalizing levels of downstream target genes that may play a role in CAVD pathogenesis. Strategies to increase miR binding specificity to target mRNAs include the utilization of locked nucleic acids or 2′-O-methylation of the antisense oligonucleotides, while circulation time and cellular uptake of miRNAs can be enhanced by cholesterol conjugation (216). miR-204 expression is significantly dysregulated in CAVD and aortic stenosis, as are the miR-34 family members that are known to suppress BMP2; making these attractive miR-based therapeutic targets (137, 163, 165, 183, 217). In addition, LncRNA H19 promotes osteogenic-like changes in VICs by silencing NOTCH1. Given its success as a therapeutic strategy for pancreatic cancer (218), LncRNA H19 may also serve as potential CAVD therapy (160).

While a handful of genes have been identified largely in mice, as playing a causative role in CAVD, manipulating gene function therapeutically to attenuate calcific nodule progression in humans is challenging, largely due to the off-target effects in non-valvular structures. In susceptible mouse models, pharmacologically targeting enzymes important in signaling pathways increased in calcified valves has been successful in promoting the regression or reducing the formation of nodules, including Carbonic Anhydrase (219) and Cyclooxygenase 2 (116), respectively. As discussed here, the epigenetic regulation of CAVD is evolving and miRNA- or LncRNA-based therapeutics to target key drivers of pathogenesis could potentially be beneficial in restoring the expression of affected genes. In addition to genes, risk factors for CAVD are also environmental, or influenced by life style habits. Interestingly, while CAVD is observed in old, hypercholesterolemic mice (220), studies to increase exercise and reduce dietary cholesterol intake did not influence aortic valve disease (221), although lowering plasma cholesterol genetically did alleviate calcific nodule formation, but not stenosis (222). Thus, once end-stage calcification and stenosis occur, eliminating the cause/risk-factor for CAVD may not abrogate the disease, supporting the notion that CAVD is irreversible and early intervention is needed.

In summary, current therapies for the treatment of CAVD are limiting and long-term ineffective. Moving forward there is a critical need to increase our understanding of the etiology of CAVD, and the molecular and cellular process that contribute to onset and progression of calcific nodule formation. From this we will be poised to advance the discovery of mechanistic-based therapies that treat the underlying cause, rather than just the symptoms.

## Author contributions

VM and JL wrote the text. JL generated figures and edited the final submission.

### Conflict of interest statement

The authors declare that the research was conducted in the absence of any commercial or financial relationships that could be construed as a potential conflict of interest.
